# Superior long-term survival with acceptable safety of ATG/G-CSF–based haplo-HSCT with intensified BU+MEL/TT conditioning in CR Pediatric Non-DS–AMKL

**DOI:** 10.3389/fonc.2026.1741238

**Published:** 2026-04-30

**Authors:** Fei Pan, Jing Long, Xing-yu Cao, Yue Lu, Jian-ping Zhang, Yan-li Zhao, Min Xiong, Fang-min Pan, Guan-lan Yue, Kang Gao, Zhi-jie Wei

**Affiliations:** 1 Beijing Lu Daopei Hospital, Beijing, China; 2 Beijing Lu Daopei Institute of Hematology, Beijing, China; 3 Hebei Yanda Lu Daopei Hospital, Langfang, China

**Keywords:** haplo-HSCT, intensified conditioning, long-term survival, MEL or TT, pediatric non-DS AMKL

## Abstract

**Background:**

Non–Down syndrome acute megakaryoblastic leukemia (non–DS–AMKL) is a rare but high-risk subtype of pediatric AML with dismal outcomes. Allo-HSCT in first complete remission (CR1) is recommended as the preferred strategy for improving survival, yet the benefit of BU/CY intensification with melphalan (MEL) or thiotepa (TT) remains unclear.

**Methods:**

We retrospectively analyzed 70 pediatric non–DS–AMKL patients undergoing ATG/G-CSF–based haplo-HSCT between March 2010 and February 2024, comparing standard BU/CY (n=25) with intensified BU+MEL/TT (n=45). Endpoints included OS, LFS, RI, NRM, and TRM. Survival was estimated by Kaplan–Meier and compared with the log-rank test, while cumulative incidences were analyzed using Gray’s test. Cox proportional hazards and Fine–Gray competing-risk regression models were applied for univariate and multivariate analyses, as appropriate.

**Results:**

Baseline clinical characteristics were comparable between groups, whereas graft CD34+ (median, 10.40×10^6^/kg vs 5.85×10^6^/kg) and CD3+ (median, 4.54×10^8^/kg vs 1.89×10^8^/kg) cell doses were significantly higher in the BU+MEL/TT group (both P<0.001). Median follow-up was 25 months (range, 13–91.2). Day-100 TRM was similar between groups (6.67% vs 8.00%, *P* = 0.81), with no significant differences in engraftment, GVHD, viral reactivations. No VOD/SOS or TMA events were observed. Among patients transplanted in CR, the BU+MEL/TT group demonstrated superior 5-year OS (75.2% vs 50.0%, *P* = 0.045) and LFS (76.3% vs 50.0%, *P* = 0.039), with numerically lower RI and NRM. In contrast, patients transplanted in NR/PR had poor outcomes in both groups, with 1-year OS and LFS of approximately 20%. In multivariable Cox analyses, intensified conditioning (BU+MEL/TT vs BU) improved OS (HR 0.20, P = 0.047) and LFS (HR 0.29, P = 0.034). Complex cytogenetics (OS: HR 2.38, P = 0.016; LFS: HR 2.58, P = 0.011) and pre-transplant NR/PR status (OS: HR 5.53, P = 0.001; LFS: HR 3.55, P = 0.004) were independent adverse factors. In competing-risk models, conditioning was not associated with RI or NRM, whereas NR/PR status significantly increased relapse (HR 3.39, P = 0.019) and NRM (HR 3.73, P = 0.007).

**Conclusions:**

In pediatric non–DS–AMKL, intensified conditioning (BU+MEL/TT) independently improved OS and LFS in patients transplanted in CR without increasing early NRM. Pre-transplant NR/PR status was the strongest adverse factor, markedly increasing RI and NRM, underscoring the importance of achieving remission before transplantation.

## 1 Introduction

Non–Down syndrome acute megakaryoblastic leukemia (non–DS-AMKL) is a rare and highly aggressive subtype of acute myeloid leukemia (AML), categorized as high-risk and therapy-resistant. In contrast to DS-AMKL, which is highly sensitive to chemotherapy with long-term survival rates approaching 80% (1), the overall survival (OS) of patients with non–DS-AMKL remains dismal at only 10–30% under standard chemotherapy regimens (2, 3). This poor prognosis is partly driven by recurrent fusion genes—such as *CBFA2T3::GLIS2, KMT2A rearrangements (KMT2A-r)*, and *NUP98::KDM5A*—which confer distinct molecular features and profound chemoresistance (4, 5).

Comprehensive molecular profiling has revealed substantial heterogeneity within non–DS-AMKL, underscoring the need for risk-adapted therapeutic strategies to improve survival outcomes across different molecular subgroups. Targeted therapies against *CBFA2T3::GLIS2* or chemoresistance-associated pathways are under development and may offer new therapeutic opportunities (6). The *CBFA2T3::GLIS2* fusion, in particular, is an independent high-risk factor in pediatric non–DS-AMKL, strongly associated with extremely poor survival, early relapse, and resistance to chemotherapy. Therefore, intensified therapy followed by early allogeneic hematopoietic stem cell transplantation (allo-HSCT) is recommended to improve outcomes (7). However, rapid disease progression and the limited availability of timely HLA-matched sibling or unrelated donors often delay transplantation (8).

Haploidentical HSCT (haplo-HSCT) has emerged as a practical alternative donor strategy and has demonstrated survival outcomes comparable to matched sibling or unrelated donor transplantation in high-risk pediatric AML (9). A critical question, however, remains unresolved—how does the intensity of conditioning influence post-transplant outcomes? Recent studies suggest that a decitabine-enhanced busulfan/cyclophosphamide (BU/CY) regimen should be prioritized for AML patients undergoing related donor HSCT, particularly those with intermediate- or high-risk disease, as it reduces relapse, improves survival, and is well tolerated (10). Preliminary data from our center indicated that augmenting the conventional BU/CY regimen with melphalan (MEL) or thiotepa (TT) might further improve outcomes in non–DS-AMKL. Nevertheless, high-quality comparative evidence is lacking. To address this gap, we conducted a retrospective analysis of 70 non–DS-AMKL patients who underwent ATG/G-CSF–based haplo-HSCT at our institution over the past decade. This study aimed to systematically compare the long-term efficacy and safety of standard Bu/Cy conditioning versus Bu/Cy intensified with MEL or TT.

## 2 Methods

### 2.1 Study design and patients

This retrospective study included pediatric patients with non-DS-AMKL who underwent haplo-HSCT between March 2010 and February 2024. Eligibility criteria were age <14 years at diagnosis, confirmation according to the 2022 WHO classification (11), and first haplo-HSCT. Patients with DS-AMKL or secondary AML were excluded. Patients were divided into two groups according to the finally myeloablative conditioning (MAC) regimens received: a standard BU/CY-based regimen group (BU group) and an intensified conditioning group (BU+MEL/TT group). Follow-up was completed on March 20, 2025. This study was approved by the Ethics Committee of Hebei Yanda Lu Daopei Hospital, and written informed consent was obtained from all patients or their legal guardians in accordance with the Declaration of Helsinki.

### 2.2 Conditioning regimens selection

Conditioning regimens were selected according to institutional protocols that evolved over time based on clinical outcomes and accumulated experience. During the early study period, patients with AML-M7 primarily received conventional myeloablative conditioning with busulfan and cyclophosphamide (BU/CY), which represented the institutional standard at that time. Given the relatively high relapse rate observed with this approach, intensified conditioning regimens incorporating melphalan (BU/CY+MEL) were subsequently introduced based on prior institutional experience suggesting improved outcomes in high-risk AML.

Following the reavailability of thiotepa and increasing recognition of the risk of central nervous system involvement in AML-M7, thiotepa-containing regimens were adopted as an alternative intensification strategy, particularly for patients with CNS disease or those considered at higher risk for CNS relapse.

Conditioning regimen selection was not determined solely by individual physician preference. All cases were reviewed in departmental transplant conferences, and the final regimen was determined according to institutional treatment guidelines and approved by the department director.

The BU group received cytarabine 10 g/m^2^ (days –14 to –10, once daily), busulfan 12.8 mg/kg (days –9 to –6, intravenously in divided doses), cyclophosphamide 3.6 g/m^2^ (days –5 to –4, divided doses), and antithymocyte globulin (ATG) 7.5 mg/kg (days –5 to –2). The BU+MEL/TT group received the same backbone regimen with the addition of either melphalan (110–140 mg/m^2^) or thiotepa (5–7.5 mg/kg), administered on days –3 and –2 according to institutional protocol and clinical indication. Stem cells were collected from healthy donors following granulocyte colony-stimulating factor (G-CSF) mobilization.

### 2.3 GVHD prophylaxis and post-transplant management

GVHD prophylaxis consisted of cyclosporine A (CSA) or tacrolimus (FK506) combined with mycophenolate mofetil (MMF) and short-course methotrexate (sMTX) administered on days +1, +3, +6, and +11. This platform was used as the institutional standard GVHD prophylaxis regimen for haploidentical transplantation throughout the entire study period. Prophylaxis against veno-occlusive disease (VOD) included oral ursodeoxycholic acid from conditioning initiation to day +28 post-transplant, fresh frozen plasma from conditioning start today −1, and alprostadil from day −9 before transplantation through day +28.

Post-transplant maintenance therapy was routinely administered. Beginning approximately 3 months after HSCT, patients with stable hematologic recovery received azacitidine maintenance therapy (32 mg/m^2^/day for 7 days every 28 days) for up to four cycles. Prophylactic donor lymphocyte infusion (DLI) was not routinely performed; instead, a pre-emptive strategy was adopted in which DLI was administered only upon detection of measurable residual disease (MRD), starting at 1 × 10^5^ cells/kg with escalation in the absence of GVHD. Immunomodulatory agents were used when clinically indicated to enhance graft-versus-leukemia effects under close MRD and GVHD monitoring.

### 2.4 MRD assessment

MRD was assessed by multiparameter flow cytometry (MFC) at our institutional laboratory using ≥10-color panels, with higher-parameter platforms introduced in later years. The analytical sensitivity ranged from 10^-4^ to 10^-6^ depending on disease characteristics and event acquisition. MRD positivity was primarily defined as ≥0.1% according to consensus criteria, with lower thresholds applied when supported by leukemia-associated immunophenotypes. Pre-transplant MRD evaluation was routinely performed approximately 7 days before transplantation.

### 2.5 Outcome definitions

The primary endpoints of this study were overall survival (OS), leukemia-free survival (LFS), cumulative incidence of relapse (RI), non-relapse mortality (NRM). OS was defined as the time from transplantation to death from any cause, and LFS as the time to either relapse or death. CIR was defined as hematologic relapse following HSCT, with NRM treated as a competing event. NRM was defined as death without prior relapse. Secondary endpoints included hematologic engraftment, graft-versus-host disease (GVHD), and CMV/EBV reactivation. WBC engraftment was defined as the first of three consecutive days with an absolute neutrophil count (ANC) ≥ 0.5 × 10^9^/L, and platelet engraftment as the first of seven consecutive days with a platelet count ≥ 20 × 10^9^/L without transfusion support. Acute GVHD (aGVHD) was graded according to the published criteria within the first 100 days post-HSCT, and chronic GVHD (cGVHD) was classified based on the *NIH 2014 consensus* (12–14).

### 2.6 Statistical analysis

Group comparisons were performed using the Mann–Whitney U test for continuous variables and the chi-square or Fisher’s exact test for categorical variables, as appropriate. OS and LFS were estimated using the Kaplan–Meier method and compared using the log-rank test. RI, NRM, and GVHD were analyzed using competing-risk methods, with between-group comparisons performed using Gray’s test. For the estimation of the cumulative incidence of GVHD, death and relapse without prior GVHD were treated as competing events. Univariate and multivariate analyses for OS and LFS were conducted using Cox proportional hazards regression models. For endpoints involving competing risks, regression analyses were performed using the Fine–Gray subdistribution hazards model. Variables with *P* < 0.2 in univariate analysis or considered clinically relevant were included in multivariate models. Hazard ratios (HRs) and corresponding 95% confidence intervals (CIs) were reported. All statistical analyses were performed using R software (version 4.5.0) and SPSS (version 29.0). Graphs were generated using R and GraphPad Prism (version 9.3). A two-sided *P* < 0.05 was considered statistically significant.

## 3 results

### 3.1 Patient characteristics and haplo-HSCT information

A total of 70 patients with non-DS-AMKL were included in this study and divided into two groups according to the conditioning regimen: the BU+MEL/TT group (n=45) and the BU group (n=25). Baseline characteristics were generally comparable (**Table 1**). No significant differences were observed in recipient sex, age, WBC count, hemoglobin, platelet count, bone marrow blast percentage, fusion gene status, cytogenetics, or pre-transplant status (all *P* > 0.05). Most patients (97%) received combined BM+PB grafts. However, the BU+MEL/TT group received significantly higher doses of CD34^+^ cells (median 10.40 × 10^6^/kg vs. 5.85 × 10^6^/kg, *P* < 0.001), and CD3^+^ cells (median 4.54 × 10^8^/kg vs. 1.89 × 10^8^/kg, *P* < 0.001). The distribution of HSCT years was comparable between the two groups (*P* = 0.43), with similar proportions of patients undergoing HSCT during 2015–2018, 2019–2021, and 2022–2024.

**Table 1:** Patient characteristics and haplo-HSCT information.

Characteristics	BU+MEL/TT group (n=45)	BU group (n=25)	*p-value*
Recipient sex; n (%)			*0.45*
Male	24 (53.3)	11 (44)	
Female	21 (46.7)	14 (56)	
Recipient age; median (range) years	2.4 (0.9-9.4)	2.8 (1.3-9.9)	*0.07*
Donor age; median (range) years	33 (9-43)	31 (17-52)	*0.63*
Time from diagnosis to HSCT, median (range), months	5.6 (1.8-18.4)	6.7 (2.9-24.1)	*0.12*
WBC count at diagnosis, ×10^9^/L	13.8 (10.2-621.8)	12.6 (2.4-75.8)	*0.54*
Hemoglobin level at diagnosis, g/L	87.0 (9.0-124.0)	81.0 (2.8-121.0)	*0.30*
PLT count at diagnosis, ×10^9^/L	21.0 (2.0-293.0)	27.0 (4.0-273.0)	*0.79*
BM blast percentage at diagnosis, %	40.1 (4.6-94.0)	42.0 (4.8-99.0)	*0.91*
Fusion genes; n (%)			*0.13*
Positive	21 (46.7)	7 (28)	
KMT2A-r	6 (13.3)	4 (16)	
CBFA2T3::GLIS2	7 (15.7)	2 (8)	
NUP98::KDM5A	2 (4.4)	0	
* RBM15::MKL1*	2 (4.4)	0	
* Other fusion genes*	4 (8.9)	1 (4)	
Negative	24 (53.3)	18 (72)	
Complex cytogenetics; n (%)			*0.45*
Yes	14 (31.1)	10 (40)	
No	31 (68.9)	15 (60)	
Stem cell source; n (%)			*1.00*
BM+PB	44 (97.8)	24 (96)	
PB	1 (2.2)	1 (4)	
GVHD prophylaxis regimen; n (%)			*0.66*
CsA-based	42 (93.3)	22 (88)	
FK506-based	3 (6.7)	3 (12)	
HLA matched; n (%)			*0.45*
5/10	36 (80.0)	18 (72)	
≥6/10	9 (20.0)	7 (28)	
Pre-transplant status; n (%)			*0.36*
CR-MRD-	3 (6.7)	4 (16)	
CR-MRD+	28 (62.2)	16 (64)	
NR/PR	14 (31.1)	5 (20)	
Haploidentical grafts; median (range)			
CD34^+^cells,×106/kg	10.4 (4.1-29.3)	5.9 (3.8-20.0)	< 0.001
CD3^+^ cells,×108/kg	4.5 (1.4-19.4)	1.9 (0.6-7.9)	< 0.001
Year of HSCT; n (%)			0.43
2015-2018	8 (17.8)	8 (32.0)	
2019-2021	24 (53.3)	12 (48.0)	
2022-2024	13 (28.9)	5 (20.0)	

BM, bone marrow; BU, busulfan; CI, confidence interval; CR, complete remission; NR, non-remission; PR, partial remission; CsA, cyclosporine A; FK506, tacrolimus; GVHD, graft-versus-host disease; HLA, human leukocyte antigen; HSCT, hematopoietic stem cell transplantation; MEL, melphalan; TT, thiotepa; MNC, mononuclear cells; MRD, minimal residual disease; PB, peripheral blood; PLT, platelet; WBC, white blood cell.

### 3.2 Engraftment and hematologic recovery

Neutrophil and platelet engraftment rates were comparable between groups. By day 28, neutrophil engraftment was achieved in 97.8% of patients in the BU+MEL/TT group and 96.0% in the BU group (*P* = 1.00). Platelet engraftment by day 100 occurred in 91.1% and 88.0% of patients, respectively (*P* = 0.69). The median time to neutrophil recovery was 12 days in the BU+MEL/TT group versus 14 days in the BU group (*P* = 0.07), while median platelet recovery times were 9 and 11 days, respectively (*P* = 0.22). Although not statistically significant, hematopoietic recovery tended to occur earlier in the BU+MEL/TT group.

### 3.3 Graft-versus-host disease and viral reactivations

The incidence of acute and chronic GVHD appeared relatively high in this non-DS-AMKL cohort. The cumulative incidence of grade II–IV aGVHD by day 100 was comparable between groups (60.12% vs. 60.00%, *P* = 0.24), as was the incidence of grade III–IV aGVHD (42.31% vs. 40.00%, *P* = 0.61). cGVHD occurred more frequently in the BU+MEL/TT group than in the BU group (51.46% vs. 28.00%, *P* = 0.05), with a numerically higher incidence of severe chronic GVHD (32.59% vs. 12.00%, *P* = 0.07).

In univariable analyses, no variables were significantly associated with grade III–IV aGVHD or severe cGVHD (**Table 2**). The conditioning regimen was not associated with severe aGVHD (MEL/TT+BU vs BU: HR 0.83, 95% CI 0.41–1.69, *P* = 0.610), and no independent risk factors were identified. For severe cGVHD, the MEL/TT+BU regimen showed a nonsignificant trend toward increased risk in univariable analysis (HR 2.97, 95% CI 0.85–10.39, *P* = 0.088), which was not retained after adjustment (HR 2.28, 95% CI 0.51–10.29, *P* = 0.280). A higher CD34^+^ cell dose (>8 ×10^6^/kg) demonstrated a borderline association in univariable analysis (HR 2.68, 95% CI 0.96–7.49, *P* = 0.061) but was not significant in multivariable analysis (HR 2.09, 95% CI 0.74–5.91, *P* = 0.170).

**Table 2:** Univariable and multivariable analyses of risk factors for grade III–IV aGVHD and severe cGVHD.

Characteristic	III-IV Grade aGVHD				Severe cGVHD			
	Univariable		Multivariable		Univariable		Multivariable	
	HR (95% CI)	*p-value*	HR (95% CI)	*p-value*	HR (95% CI)	*p-value*	HR (95% CI)	*p-value*
Conditioning regimen								
BU	Reference				Reference		Reference	
MEL/TT+BU	0.83 (0.41-1.69)	0.610			2.97 (0.85-10.39)	0.088	2.28 (0.51-10.29)	0.280
High-risk fusion gene								
No	Reference		Reference		Reference			
Yes	0.42 (0.17-1.02)	0.056	0.39 (0.14-1.08)	0.071	0.87 (0.31-2.44)	0.780		
Complex cytogenetics								
No	Reference				Reference		Reference	
Yes	0.86 (0.41-1.78)	0.680			1.90 (0.75-4.85)	0.180	1.73 (0.65-4.65)	0.270
Recipient age,year								
≤2	Reference		Reference		Reference			
>2	1.88 (0.80-4.39)	0.150	1.91 (0.55-6.68)	0.310	1.25 (0.44-3.51)	0.680		
Pre-transplant status								
CR-MRD-	Reference				Reference			
CR-MRD+	2.15 (0.66-6.98)	0.200			1.17 (0.23-5.97)	0.850		
NR/PR	1.85 (0.88-3.86)	0.100			0.55 (0.16-1.86)	0.340		
Donor_age								
≤30	Reference				Reference			
>30	1.16 (0.57-2.38)	0.680			1.17 (0.44-3.10)	0.750		
Donor_gender								
Male	Reference		Reference		Reference			
Female	1.76 (0.85-3.64)	0.130	1.25 (0.56-2.87)	0.590	0.52 (0.15-1.82)	0.310		
GVHD prophylaxis regimen								
CSA-based	Reference				Reference		Reference	
FK-506 based	1.16 (0.38-3.54)	0.800			2.45 (0.78-7.69)	0.120	2.48 (0.77-8.02)	0.130
HLA matched								
5/10	Reference		Reference		Reference			
>5/10	1.69 (0.76-3.70)	0.190	1.60 (0.70-3.63)	0.270	0.65 (0.19-2.16)	0.480		
CD34+cells,×106/kg								
≤8	Reference		Reference		Reference		Reference	
>8	0.58 (0.29-1.15)	0.120	0.61 (0.12-3.11)	0.550	2.68 (0.96-7.49)	0.061	2.09 (0.74-5.91)	0.170
CD3+ cells,×108/kg								
≤3	Reference				Reference		Reference	
>3	0.70 (0.35-1.42)	0.330			2.55 (0.85-7.65)	0.094	1.08 (0.28-4.21)	0.920

aGVHD, acute graft-versus-host disease; BU, busulfan; CI, confidence interval; CMV, cytomegalovirus; cGVHD, chronic graft-versus-host disease; MEL, melphalan; TT, thiotepa.

The CMV reactivation rates by day 100 were comparable (40.08% vs. 44.00%, *P* = 0.48), as were EBV reactivation rates (15.56% vs. 12.00%, *P* = 0.68). Other conditioning related toxicities included grade II–III oral mucositis in approximately 25% of patients. No cases of veno-occlusive disease/sinusoidal obstruction syndrome (VOD/SOS) were observed. In addition, no transplant-associated thrombotic microangiopathy (TMA) events occurred during the study period, corresponding to an incidence of 0%.

### 3.4 Long-term outcomes

The median follow-up time was 25 months (range, 13–91.2), a total of 33 patients succumbed, with 94% (31/33) of deaths occurring within the first year post-transplant—predominantly in patients who underwent transplantation in NR/PR status. Among the 37 surviving patients, the median follow-up duration was 50.8 months (range, 2.6–91.2). Day-100 NRM occurred in five patients. The causes included sepsis (n=1), gastrointestinal bleeding leading to hemorrhagic shock (n=2), and severe intestinal GVHD (n=1). The day-100 NRM rate was comparable between the two groups (6.67% vs. 8.00%, *P* = 0.81).

**Figure 1:**
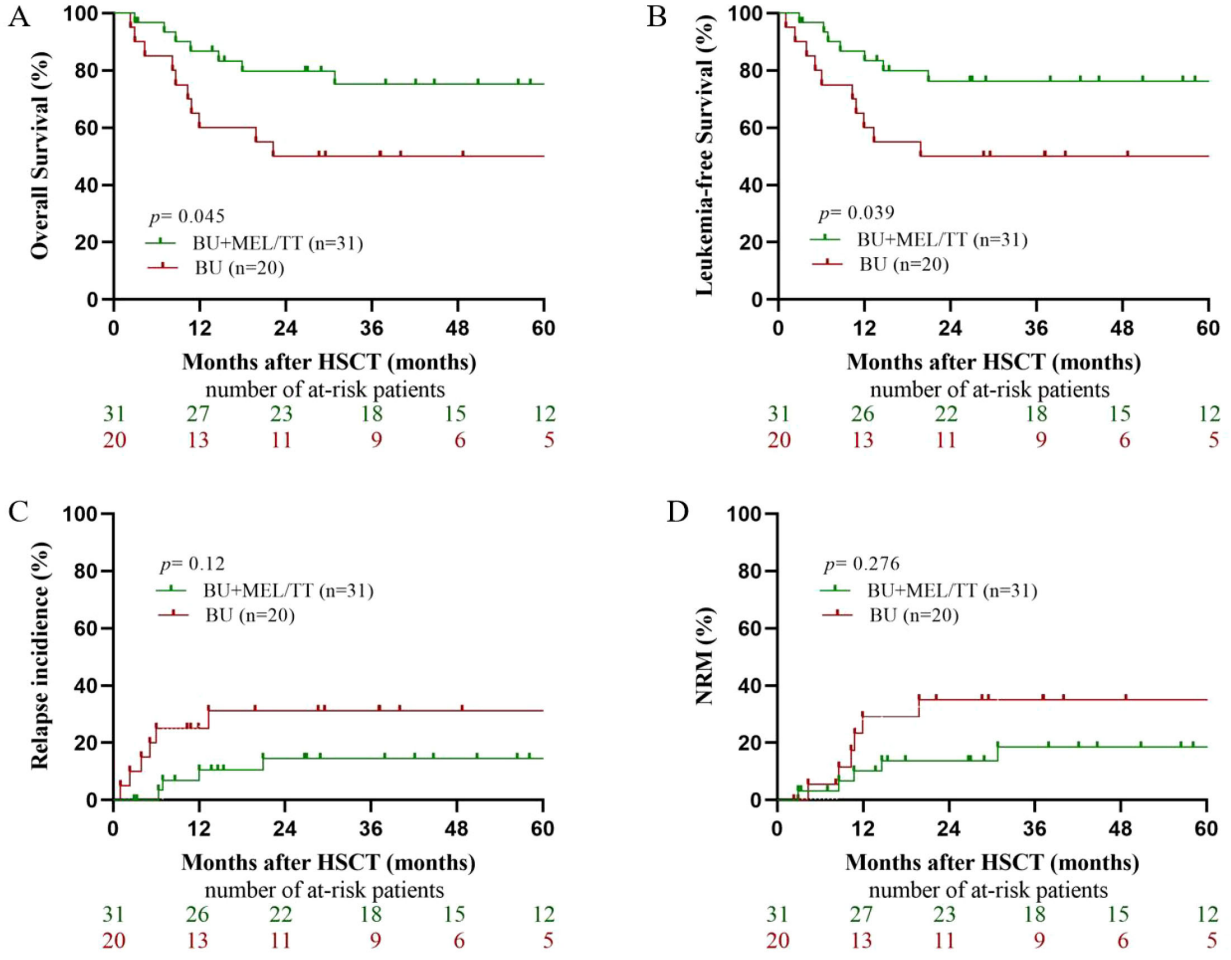
Long-term outcomes of CR non-DS-AMKL patients. **(A)** displays overall survival (OS) between the two groups; **(B)** shows leukemia-free survival (LFS); **(C)** presents the cumulative incidence of relapse; and **(D)** illustrates non-relapse mortality (NRM).

Among patients who achieved CR prior to transplantation, the BU+MEL/TT group demonstrated significantly superior outcomes compared with the BU group (**Figure 1**). The 5-year OS was 75.24% (95% CI, 59.11–91.38) vs. 50% (95% CI, 28.09–71.91; *P* = 0.045), and the 5-year LFS was 76.32% (95% CI, 60.94–91.69) vs. 50% (95% CI, 28.09–71.91; *P* = 0.039). Although not statistically significant, both 5-year RI and 5-year NRM were numerically lower in the BU+MEL/TT group (RI: 13.65% vs. 30.36%, *P* = 0.12; NRM: 17.8% vs. 30%, *P* = 0.276).

**Figure 2:**
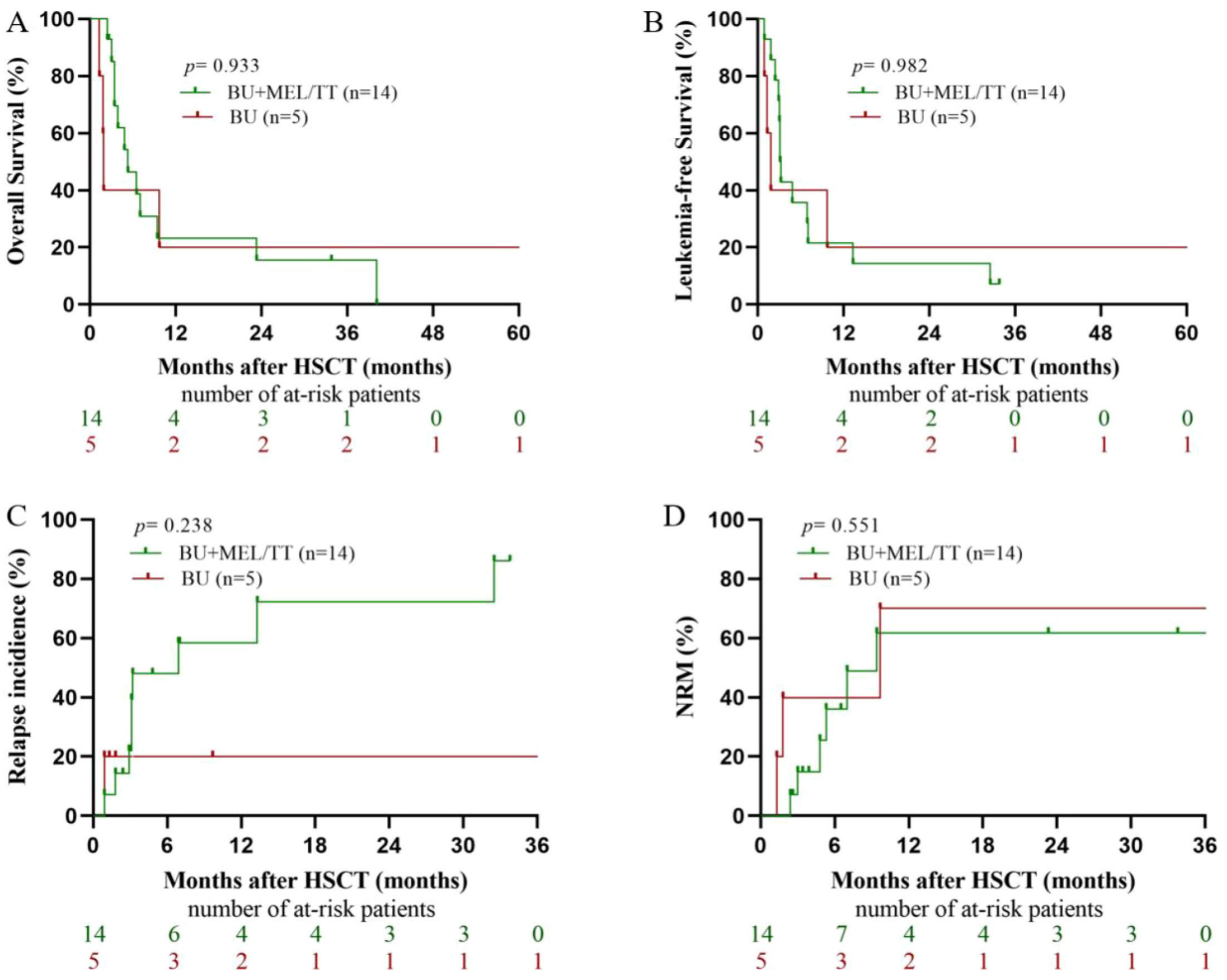
Long-term outcomes of NR/PR non-DS-AMKL patients. **(A)** displays overall survival (OS) between the two groups; **(B)** shows leukemia-free survival (LFS); **(C)** presents the cumulative incidence of relapse; and **(D)** illustrates non-relapse mortality (NRM).

In contrast, among patients transplanted in NR/PR status, survival outcomes remained poor in both groups (**Figure 2**). The 1-year OS was 23.21% (95% CI, 0.2–42.61) in the BU+MEL/TT group vs. 20% (95% CI, 0–55.06) in the BU group (*P* = 0.933), and the 1-year LFS was 21.43% (95% CI, 0–42.92) vs. 20% (95% CI, 0–55.06; *P* = 0.982). The 1-year RI was higher in the BU+MEL/TT group (57.14% vs. 20.00%, *P* = 0.238), while the 1-year NRM remained comparable (45.83% vs. 60.00%, *P* = 0.551).

### 3.5 Univariable and multivariable analyses of prognostic outcomes

In Cox proportional hazards regression analyses for OS and LFS (**Table 3**), the MEL/TT+BU conditioning regimen was independently associated with improved survival outcomes compared with BU alone, demonstrating superior OS (HR 0.20, 95% CI 0.04–0.96, *P* = 0.047) and LFS (HR 0.29, 95% CI 0.19–0.87, *P* = 0.034). In contrast, complex cytogenetics remained an independent adverse prognostic factor for both OS (HR 2.38, 95% CI 1.18–4.79, *P* = 0.016) and LFS (HR 2.58, 95% CI 1.23–5.37, *P* = 0.011). Pre-transplant disease status strongly influenced outcomes, with patients transplanted in NR/PR demonstrating significantly inferior OS (HR 5.53, 95% CI 2.64–11.59, *P* = 0.001) and LFS (HR 3.55, 95% CI 1.49–8.44, *P* = 0.004) compared with those in CR-MRD−. No significant associations were observed for high-risk fusion genes, recipient age, donor characteristics, GVHD prophylaxis, HLA matching, or HSCT-year, infused CD34^+^ and CD3^+^ cell doses.

**Table 3:** Cox proportional hazards regression analysis for OS and LFS.

Characteristic	OS				LFS			
	Univariable		Multivariable		Univariable		Multivariable	
	HR (95% CI)	*p-value*	HR (95% CI)	*p-value*	HR (95% CI)	*p-value*	HR (95% CI)	*p-value*
Conditioning regimen								
BU	reference		reference		reference		reference	
MEL/TT+BU	0.38 (0.14-0.99)	*0.042*	0.20 (0.04-0.96)	*0.047*	0.33 (0.27-0.97)	*0.040*	0.29 (0.19-0.87)	*0.034*
High-risk fusion gene								
No	reference				reference		reference	
Yes	0.60 (0.27-1.34)	*0.2152*			0.57 (0.26-1.26)	*0.167*	0.52 (0.23-1.17)	*0.116*
Complex cytogenetics								
No	reference		reference		reference		reference	
Yes	2.60 (1.31-5.18)	*0.0064*	2.38 (1.18-4.79)	*0.016*	2.85 (1.45-5.61)	*0.003*	2.58 (1.23-5.37)	*0.011*
Recipient age,year								
≤2	reference				reference			
>2	0.85 (0.42-1.73)	*0.6519*			0.85 (0.42-1.73)	*0.661*		
Pre-transplant status								
CR-MRD-	reference		reference		reference		reference	
CR-MRD+	1.48 (0.43-5.17)	*0.5356*	1.68 (0.48-5.90)	*0.419*	1.49 (0.43-5.20)	*0.530*	1.10 (0.32-3.79)	*0.881*
NR/PR	5.81 (2.79-12.10)	*0.001*	5.53 (2.64-11.59)	*0.001*	6.20 (3.00-12.8)	*0.001*	3.55 (1.49-8.44)	*0.004*
Donor_age								
≤30	reference				reference			
>30	1.10 (0.54-2.24)	*0.7877*			1.13 (0.56-2.29)	*0.729*		
Donor_gender								
Male	reference				reference		reference	
Female	0.60 (0.26-1.38)	*0.2279*			0.57 (0.25-1.30)	*0.179*	0.71 (0.28-1.76)	*0.453*
GVHD prophylaxis regimen								
CSA-based	reference				reference			
FK-506 based	1.31 (0.46-3.73)	*0.6157*			1.27 (0.45-3.63)	*0.650*		
HLA matched								
5/10	reference				reference			
>5/10	0.82 (0.35-1.88)	*0.6319*			0.77 (0.34-1.78)	*0.547*		
CD34+cells,×106/kg								
≤8	reference				reference			
>8	0.84 (0.42-1.67)	*0.6193*			0.89 (0.45-1.75)	*0.739*		
CD3+ cells,×108/kg								
≤3	reference				reference			
>3	0.79 (0.39-1.57)	*0.4961*			0.81 (0.41-1.61)	*0.547*		
HSCT year								
2015-2018	reference				reference			
2019-2021	0.59 (0.27-1.31)	*0.199*			0.62 (0.28-1.37)	*0.237*		
2022-2024	0.66 (0.25-1.74)	*0.401*			0.70 (0.27-1.77)	*0.450*		

In Fine–Gray competing-risk regression analyses for RI and NRM (**Table 4**), the conditioning regimen was not associated with RI or non-relapse mortality. Complex cytogenetics showed an increased risk of relapse in univariable analysis (HR 2.92, 95% CI 1.24–6.88, *P* = 0.015), with a borderline association retained after adjustment (HR 2.54, 95% CI 0.93–6.92, *P* = 0.069). Pre-transplant disease status remained the strongest prognostic factor, as patients transplanted in NR/PR had significantly higher risks of both relapse (HR 3.39, 95% CI 1.26–9.41, *P* = 0.019) and NRM (HR 3.73, 95% CI 1.43–9.71, *P* = 0.007) compared with those in CR-MRD−. A higher CD3^+^ cell dose (>3 ×10^8^/kg) showed a borderline association with reduced relapse risk in univariable analysis (HR 0.46, 95% CI 0.19–1.09, *P* = 0.077), which was not maintained in multivariable analysis. No significant associations were observed for high-risk fusion genes, recipient age, donor characteristics, GVHD prophylaxis, HLA matching, CD34^+^ cell dose or HSCT-year.

**Table 4:** Fine–gray regression analysis of risk factors for RI and NRM.

Characteristic	RI				NRM			
	Univariable		Multivariable		Univariable		Multivariable	
	HR (95% CI)	*p-value*	HR (95% CI)	*p-value*	HR (95% CI)	*p-value*	HR (95% CI)	*p-value*
Conditioning regimen								
BU	Reference				Reference			
MEL/TT+BU	1.01 (0.40-2.52)	*0.990*			0.68 (0.28-1.60)	*0.370*		
High-risk fusion gene								
No	Reference		Reference		Reference			
Yes	0.47 (0.16-1.39)	*0.170*	0.76 (0.17-1.77)	*0.340*	0.71 (0.25-1.97)	*0.510*		
Complex cytogenetics								
No	Reference		Reference		Reference		Reference	
Yes	2.92 (1.24-6.88)	*0.015*	2.54 (0.93-6.92)	*0.069*	1.85 (0.78-4.41)	*0.170*	1.79 (0.72-4.45)	*0.211*
Recipient age,year								
≤2	Reference				Reference			
>2	0.70 (0.29-1.68)	*0.420*			1.20 (0.46-3.10)	*0.710*		
Pre-transplant status								
CR-MRD-	Reference		Reference		Reference		Reference	
CR-MRD+	1.66 (0.39-7.32)	*0.500*	1.08 (0.21-5.66)	*0.930*	2.71 (0.80-9.16)	*0.110*	2.97 (0.92-9.64)	*0.069*
NR/PR	3.78 (1.53-9.39)	*0.004*	3.39 (1.26-9.41)	*0.019*	3.90 (1.49-10.16)	*0.005*	3.73 (1.43-9.71)	*0.007*
Donor_age								
≤30	Reference				Reference			
>30	1.10 (0.44-2.75)	*0.830*			1.59 (0.63-4.01)	*0.330*		
Donor_gender								
Male	Reference				Reference			
Female	0.63 (0.21-1.88)	*0.410*			0.54 (0.19-1.47)	*0.230*		
GVHD prophylaxis regimen								
CSA-based	Reference				Reference			
FK-506 based	0.48 (0.08-3.11)	*0.440*			1.85 (0.64-5.36)	*0.250*		
HLA matched								
5/10	Reference				Reference			
>5/10	1.11 (0.42-2.92)	*0.840*			0.77 (0.27-2.19)	*0.620*		
CD34+cells,×106/kg								
≤8	Reference				Reference			
>8	1.08 (0.45-2.58)	*0.860*			0.88 (0.37-2.09)	*0.780*		
CD3+ cells,×108/kg								
≤3	Reference		Reference		Reference			
>3	0.46 (0.19-1.09)	*0.077*	0.81 (0.24-2.71)	*0.731*	1.25 (0.49-3.13)	*0.640*		
HSCT year								
2015-2018	reference				reference			
2019-2021	0.77 (0.29-2.06)	*0.605*			0.67 (0.22-1.99)	*0.469*		
2022-2024	0.30 (0.06-1.47)	*0.136*			1.07 (0.33-3.54)	*0.906*		

## 4 Discussion

Non–DS-AMKL is a rare and highly aggressive AML subtype with poor outcomes. Standard treatment involves intensive chemotherapy to induce CR, followed by allo-HSCT improves OS from approximately 30% to nearly 50% (15), post-transplant relapse remains the leading cause of treatment failure, highlighting the need for optimized conditioning regimens and post-remission strategies. Here, we report the largest single-center cohort to date and the first study to evaluate an intensified myeloablative conditioning regimen within the ATG/G-CSF–based haplo-HSCT (Beijing protocol), in which melphalan or thiotepa is added to a BU backbone (BU+MEL/TT). This strategy was designed to deepen leukemia clearance. We compared its safety and long-term efficacy with the conventional BU/CY regimen in this high-risk, chemotherapy-resistant population.

The long-term outcomes observed in this study are encouraging, particularly among patients who achieved CR prior to haplo-HSCT. In this subgroup, the intensified BU+MEL/TT conditioning regimen significantly improved 5-year OS and LFS compared with the conventional BU/CY regimen (OS: 75.24% vs. 50%, *P* = 0.045; LFS: 76.32% vs. 50%, *P* = 0.039). Although the 5-year RI and NRM tended to be lower in the BU+MEL/TT group (RI: 13.65% vs. 30.36%, *P* = 0.12; NRM: 17.8% vs. 30%, *P* = 0.276), these differences did not reach statistical significance, likely due to limited sample size. The approximately 50% 5-year OS and LFS achieved in the BU/CY group are consistent with outcomes reported by Huang et al. in non–DS-AMKL patients undergoing haplo-HSCT in CR (16), indicating good external comparability and reinforcing the reliability of our findings. This may be attributed to the synergistic cytotoxicity of melphalan and thiotepa. Both agents exert potent anti-leukemic activity against the megakaryoblastic subtype and are effective in eradicating minimal residual disease. Melphalan induces inter- and intra-strand DNA crosslinks by binding to the N7 position of guanine, leading to double-strand breaks, disruption of DNA replication and transcription, and apoptosis. Thiotepa, metabolized to TEPA, also alkylates DNA at the N7 guanine position, forming monoadducts and crosslinks, impairing DNA repair and inducing cell death. Its high lipophilicity allows penetration of the blood–brain barrier, making it particularly beneficial in patients with CNS involvement or high CNS relapse risk, whereas melphalan offers stronger myeloablative intensity for bone marrow–dominant disease (17). Notably, the incidence of mucosal, hepatic, and renal toxicities was comparable between groups and remained manageable with current supportive care, indicating acceptable tolerability. By achieving deeper leukemic clearance, the intensified regimen may facilitate more effective graft-versus-leukemia immune responses and stable immune reconstitution post-transplant, thereby reducing relapse risk without significantly increasing transplant-related toxicity. These findings provide meaningful evidence to support optimizing conditioning intensity for CR non–DS-AMKL patients undergoing haplo-HSCT.

In contrast, patients who underwent salvage haplo-HSCT without achieving complete remission (i.e., NR/PR or MRD-positive status) experienced dismal outcomes regardless of conditioning intensity. In this cohort, both the conventional BU/CY and intensified BU+MEL/TT regimens yielded poor 1-year survival outcomes, with OS rates below 25% (20.0% vs. 23.21%, *P* = 0.933) and LFS rates similarly low (20.0% vs. 21.43%, *P* = 0.982). The 1-year RI and NRM both exceeded 20% and 45%, respectively, with no significant differences between regimens. These findings highlight the limited benefit of simply intensifying conditioning for non–DS-AMKL patients harboring high leukemic burden and chemoresistance at transplantation. Future strategies should therefore prioritize deeper cytoreduction and MRD clearance—such as novel chemotherapeutic combinations, targeted agents, or immunotherapies—to facilitate transition to a true remission state and create an optimal window for transplantation.

Given the intensified cytotoxicity of melphalan or thiotepa, concerns regarding NRM have been widely discussed. In our study, neutrophil and platelet engraftment rates were comparable between the BU+MEL/TT and BU groups (WBC: 97.8% vs. 96%; PLT: 91.1% vs. 88%), and median times to engraftment showed a non-significant trend toward faster recovery with intensified conditioning (neutrophils: 12 vs. 14 days; platelets: 9 vs. 11 days). A total of five patients experienced TRM within 100 days, including three in the BU+MEL/TT group (6.7%) and two in the BU group (8%), demonstrating no significant increase in early mortality with intensified conditioning. This acceptable safety profile may be attributable to careful dose selection—79% of patients received a total MEL dose ≤110 mg/m^2^, and only 19% received 140 mg/m^2^; TT was administered at 5–7.5 mg/kg. With ongoing patient accrual, further analyses will evaluate dose–response relationships and comparative efficacy and toxicity between melphalan- and thiotepa-based regimens. No cases fulfilling standard diagnostic criteria for VOD were identified, possibly reflecting comprehensive prophylaxis and vigilant hepatic monitoring, including serial liver function assessment and prompt abdominal ultrasonography.

Multivariate analysis confirmed that intensified conditioning (BU+MEL/TT vs BU) independently improved OS (HR 0.20, 95% CI 0.04–0.96, *P* = 0.047) and LFS (HR 0.29, 95% CI 0.19–0.87, *P* = 0.034). Complex cytogenetics adversely affected OS (HR 2.38, *P* = 0.016) and LFS (HR 2.58, *P* = 0.011), while pre-transplant NR/PR status was strongly associated with inferior OS (HR 5.53, *P* = 0.001) and LFS (HR 3.55, *P* = 0.004), supporting the survival advantage of MEL- or TT-based conditioning. Pre-transplant CR and MRD negativity remained the strongest determinants of outcome, while relapse predominantly occurred in patients with NR/PR status, MRD positivity, or high-risk cytogenetics (18). For NR/PR or MRD-positive CR patients, we explored azacitidine as a pre-transplant bridging therapy to enhance disease clearance. Although sample size is limited, early results suggest that azacitidine combined with intensified conditioning may benefit MRD-positive CR patients and merits further investigation.

Despite reducing post-transplant relapse to 13.6% among patients in CR, relapse remains a major limitation to improving outcomes in non–DS-AMKL, particularly in those undergoing transplantation with NR/PR status. This high relapse risk is largely attributable to the biological features of this leukemia subtype, including strong dormancy, immune evasion, and chemoresistance, which enable leukemic cells to persist as minimal residual disease and rapidly regrow after allo-HSCT (4). Therefore, strategies to further reduce early relapse and sustain post-transplant remission remain an urgent unmet need (6).

Novel therapeutic approaches are being actively explored. *CBFA2T3::GLIS2*–positive AML represents a unique, infant-specific molecular entity characterized by: (1) molecular sufficiency—a single fusion driving leukemogenesis without cooperating mutations; (2) distinct clinical phenotype—early infancy onset, high misdiagnosis rate, and dismal prognosis; and (3) therapeutic opportunity—emerging antibody–drug conjugates (ADCs) may overcome resistance barriers, with early trials showing encouraging activity (19). Transcriptomic profiling has revealed activation of the BMP–HOX pathway as a central feature in this subtype, highlighting the need for RNA-sequencing–based diagnosis and pathway-directed treatment. A proposed therapeutic framework includes: frontline ATRA-based epigenetic therapy plus intensive chemotherapy followed by HSCT; and for relapse, BMP inhibitors or CAR-T therapy as a bridge to second transplantation. Over the next decade, treatment goals aim to increase survival from 15–30% to >60% through an integrated “molecular classification–targeted therapy–immunotherapy” paradigm (20).

Combination azacitidine and venetoclax has shown short-term efficacy in *CBFA2T3::GLIS2*-associated AML, offering a potential therapeutic alternative (21). DOT1L and Menin inhibitors have demonstrated synergy in *MLL-rearranged* AML and may be applicable to other high-risk AMKL subtypes (22, 23). Additionally, multicenter studies on neonatal AMKL with specific cytogenetic aberrations have shown improved transplant outcomes, underscoring the importance of precise risk stratification and individualized therapy (24). Cytogenetically, our multivariate analysis identified complex karyotypes as an independent adverse factor for both OS and LFS. This observation aligns with the largest international pediatric AMKL cohort, which classified risk based on cytogenetics: favorable (*7p* abnormalities), adverse (normal karyotype, *−7, t(9;11), 9p, −13/13q–, −15*), and intermediate (others) (25). These findings emphasize that molecular and cytogenetic profiling is essential for accurate diagnosis, risk stratification, and therapeutic decision-making (3, 26). Complex karyotype (CK) and monosomal karyotype (MK) are among the strongest predictors of poor outcomes following allo-HSCT in AML, associated with >50% reduction in survival and markedly increased relapse risk. Prognostic modeling has shown that CK with MK confers the worst outcomes, highlighting the need for intensified or novel treatment strategies (27). A notable case in our cohort is the first report of a rare three-way translocation *t(1;7;22)(p13;q21;q13)*, a variant of the classic *t(1;22)*, in neonatal AMKL. This rearrangement likely drives leukemogenesis through mechanisms similar to *RBM15::MKL1* fusion and further underscores the importance of molecular diagnostics for accurate disease classification and treatment planning (28).

The incidence of aGVHD in our cohort was relatively high. Although PTCy- and TCRαβ-depletion–based strategies have been associated with lower GVHD rates (29, 30), PTCy may increase relapse risk, which is particularly concerning in AML-M7 given its aggressive and relapse-prone biology. Intensified myeloablative approaches incorporating melphalan or thiotepa may help reduce relapse, though further validation is needed. While TCRαβ-depletion offers markedly reduced GVHD and less prolonged immunosuppression, its high cost currently limits broader application. Optimization of conditioning strategies should aim to balance relapse prevention and GVHD control. This study reflects outcomes of haplo-HSCT for non-DS-AMKL at our center. Given the observed GVHD incidence, MSD or MUD may remain preferable in patients in complete remission when available.

Non-DS-AMKL carries a high post-transplant relapse risk. To reduce relapse, azacitidine maintenance was administered monthly for four cycles starting from day +100 in patients with adequate recovery. MRD re-emergence or increasing disease burden prompted chemotherapy combined with donor lymphocyte infusion (DLI), initiated at low cell doses and escalated cautiously. Immunomodulatory agents were used to enhance graft-versus-leukemia effects, with serial MRD monitoring guiding subsequent treatment.

This study has several limitations. First, it is a retrospective, single-center analysis with a relatively small cohort, which may limit the statistical power of subgroup comparisons. Second, the intensified conditioning regimen (BU+MEL/TT) was not randomly assigned, and heterogeneity in drug selection and dosing may have introduced potential bias. Third, busulfan therapeutic drug monitoring (TDM) was implemented at our center only in recent years and was available for a very limited number of patients in this cohort, precluding a meaningful analysis of its potential impact on transplant outcomes. Therefore, larger prospective multicenter studies are warranted to confirm these findings and further optimize conditioning strategies for non–DS-AMKL.

## 5 Conclusion

In this largest single-center cohort of pediatric non–DS–AMKL treated with ATG/G-CSF–based haplo-HSCT, intensifying a BU/CY backbone with MEL or TT significantly improved long-term survival in patients transplanted in CR, without increasing early TRM. Intensified conditioning and pre-transplant CR remained independent favorable factors. In contrast, outcomes for NR/PR patients remained poor. Future studies should focus on achieving MRD negativity CR before HSCT and prospectively refining conditioning intensity and drug selection to optimize outcomes in this high-risk population.

## Data Availability

The original contributions presented in the study are included in the article/supplementary material. Further inquiries can be directed to the corresponding author.
